# Meta-Analysis of 49 Roche Oncology Trials Comparing Blinded Independent Central Review (BICR) and Local Evaluation to Assess the Value of BICR

**DOI:** 10.1093/oncolo/oyad012

**Published:** 2023-03-11

**Authors:** Qinshu Lian, Jill Fredrickson, Kamila Boudier, Christiane Rothkegel, Magalie Hilton, Alexander Hillebrecht, Andrew McDonald, Na Xu

**Affiliations:** Genentech, Inc., South San Francisco, CA, USA; Genentech, Inc., South San Francisco, CA, USA; F. Hoffmann-La Roche Ltd, Basel, Switzerland; F. Hoffmann-La Roche Ltd, Basel, Switzerland; F. Hoffmann-La Roche Ltd, Basel, Switzerland; F. Hoffmann-La Roche Ltd, Basel, Switzerland; F. Hoffmann-La Roche Ltd, Basel, Switzerland; Genentech, Inc., South San Francisco, CA, USA

**Keywords:** blinded independent central review, local evaluation bias, meta-analysis, progression-free survival, oncology clinical trial, tumor response assessment

## Abstract

**Background:**

Blinded independent central review (BICR) of radiographic images is frequently conducted in oncology trials to address the potential bias of local evaluation (LE) of endpoints such as progression-free survival (PFS) and objective response rate (ORR). Given that BICR is a complex and costly process, we evaluated the agreement between LE- and BICR-based treatment effect results and the impact of BICR on regulatory decision-making.

**Materials and Methods:**

Meta-analyses were performed using hazard ratios (HRs) for PFS and odds ratios (ORs) for ORR from all randomized Roche-supported oncology clinical trials during 2006-2020 that had both LE and BICR results (49 studies with a total of over 32 000 patients).

**Results:**

Overall, the evaluation bias of LE overestimating the treatment effect compared with BICR based on PFS was numerically small and not clinically meaningful, especially for double-blind studies (HR ratio between BICR and LE: 1.044). A larger bias is more likely to occur in studies with open-label design, smaller sample sizes, or an unequal randomization ratio. The majority (87%) of the PFS comparisons led to the same statistical inference by BICR and LE. For ORR, a high degree of agreement between BICR and LE results was also observed (OR ratio of 1.065), although the agreement was slightly lower than for PFS.

**Conclusion:**

BICR did not notably impact the study interpretation nor drive the sponsor’s regulatory submission decisions. Hence, if bias can be diminished by appropriate means, LE is deemed as reliable as BICR for certain study settings.

## Teaser

Considering that blinded independent central review (BICR) of radiographic images is a complex and costly process, this study evaluated the agreement between LE- and BICR-based treatment effect results and the impact of BICR on regulatory decision-making.

**Implications for Practice**

Employing consistent guiding principles to identify scenarios in which a local evaluation assessment is sufficient to make conclusions on the treatment effect enhances the efficiency of clinical research. Our work demonstrated a limited impact of blinded independent central review (BICR) on statistical inferences and regulatory submission decisions and identified scenarios in which BICR may not be needed to control bias. In cases where BICR is needed, we suggest its implementation as a sensitivity analysis instead of as a primary endpoint. Consistent application of these principles will spare resources for the faster development of novel treatments for patients.

## Introduction

In oncology clinical trials, tumor assessments based on radiographic images are used to evaluate the anti-cancer effect of an experimental treatment. Commonly used endpoints assessed by radiographic imaging include progression-free survival (PFS) and objective response rate (ORR). PFS is defined as the time from enrollment to death or disease progression, whereas ORR is defined as the percentage of patients achieving partial or complete response during the course of a trial. Both endpoints are usually assessed according to pre-specified quantitative response criteria such as the Response Evaluation Criteria In Solid Tumours (RECISTv1.1, for solid tumors)^1^ and the Lugano classification system^2^ (for lymphoma). Application of such criteria requires the selection of lesions, the quantitative and qualitative assessment of lesions, and the identification of any new lesions over-time during the review of the images. This involves some degree of subjectivity and can, thus, introduce variability and evaluation bias.^3,4^ Note that in the remainder of this publication, we focus on the evaluation bias and refer to it simply as “bias.”

Increased variability in tumor assessments decreases the precision and the power to detect a true treatment effect, which may weaken or confound the interpretation of clinical trial results. On the other hand, bias of the treatment effect estimate is a key concern posing a threat to the validity of the results. For instance, in open-label trials the knowledge of the treatment assignment may unintentionally lead the investigator to delay calling progression in a patient on the experimental treatment or, conversely, to take a patient off an experimental treatment earlier to avoid potential side effects, especially if other treatment options exist. A double-blind design, in which investigators are blinded to the actual treatment assignment, is helpful to minimize this source of bias, however, the effect of blinding may be diminished in cases with characteristic toxicities of the experimental drug or symptomatic disease progression.

The use of blinded independent central review (BICR) of tumor assessments may reduce evaluation bias by local evaluation (LE), in particular for open-label studies or blinded studies in which complete blinding may be hard to attain.^5^ BICR may also increase precision through more uniform reader training, a smaller number of readers, and increased standardization of image evaluation. This may be especially relevant in the case of novel response criteria, rare indications, or disease presentations that are challenging to be assessed quantitatively, such as mesothelioma. Due to these advantages, Health Authorities, including the U.S. Food and Drug Administration (FDA) and European Medicines Agency (EMA), frequently tend to request BICR-derived study endpoints.

Despite these clear benefits, BICR results can also be subject to bias because of informative censoring.^4^ In addition, BICR is a very costly, resource-demanding, and time-consuming activity both for the investigator and the sponsor. Managing and monitoring the end-to-end BICR process by the sponsor requires a cross-functional team of experts including scientific functions, data management, and operational resources. The extent of involvement of each resource type depends very much on the indication, tumor assessment criteria, study complexity, and trial stage.

In event-driven trials, delay in imaging data submission or BICR reading can have an adverse impact on the statistical power and timely delivery of the study results for regulatory submissions. Furthermore, for some diseases additional clinical information besides the tumor images is needed to comprehensively assess the disease status thereby limiting the value of BICR in such cases. Several meta-analyses^4,6-9^ showed high correlation and negligible bias between treatment effect estimates from LE and BICR for PFS and/or ORR raising questions around whether BICR is truly needed and in which clinical trial scenarios.

In our present study, we expand upon these findings with meta-analyses of 49 Roche-supported randomized clinical trials with both BICR- and LE-assessed results for PFS and/or ORR. Applying statistical modeling on aggregated trial-level data, we examined the discrepancies between the BICR and LE assessments based on treatment effect estimates (hazard ratio [HR] of PFS and odds ratio [OR] of ORR). In contrast to previous similar work, this dataset included not only relatively large phase III studies in solid tumors but also smaller earlier phase studies and studies in hematological malignancies. As no inclusion criteria other than the availability of both BICR and LE assessments were applied, the dataset is not affected by any selection bias; furthermore, using only internal data, we had full access to complete datasets for each trial. We analyzed the association between some key study design factors and discrepancies between BICR and LE assessment as well as the impact of these discrepancies on binary statistical conclusions (ie, whether the difference between treatment arms was statistically significant or not). The access to the full information on each study in our database allowed us to further examine the impact of these discrepancies on subsequent regulatory submission activities.

Our findings are discussed in the context of similar analyses from other authors, and settings with a lower risk for LE bias are identified. Recommendations are provided for situations in which the conduct of BICR is not considered necessary or in which it could be limited to a sensitivity analysis to support a primary LE-based endpoint.

## Materials and Methods

The meta-analyses were performed using aggregated trial-level information from all (*N* = 49) randomized Roche-supported clinical trials during 2006-2020, regardless of the trial outcomes, in solid tumors (metastatic and early stages) and hematologic malignancies with both BICR- and LE-assessed results reported for PFS and/or ORR, respectively. All trials matching these criteria were included without any selection. The time period was selected because most trials conducted prior to 2006 used overall survival (OS) as the primary efficacy endpoint, and, thus, BICR was less commonly used in those trials. The meta-analyses performed in this paper focused on the endpoints PFS and ORR, whose definitions slightly differed across studies, however, they were consistent between BICR and LE within a study. Due to similar definitions between PFS and event-free survival (EFS), EFS results were included in the PFS meta-analysis, which allowed the assessment of broader therapeutic areas and disease stages. The following rules were applied: For hematologic malignancy studies, if both PFS and EFS results were available the one used as the primary endpoint was included in the PFS meta-analysis; if neither of them was the primary endpoint PFS results were used. For early-stage solid tumor studies, EFS (instead of PFS) results were used in the PFS meta-analysis.

Among the total of 49 studies investigated, PFS and ORR estimates were reported with both BICR- and LE-based assessments in 46 and 34 studies, respectively. Protocol pre-specified pairwise comparisons from multiple cohorts or multiple treatment arms within one study were treated as separate independent comparisons. As a result, the numbers of randomized comparisons (ie, *N* = 55 for PFS, *N* = 40 for ORR) were greater than the total number of studies.

### PFS Meta-analysis

The correlation between BICR and LE on log(HR of PFS) was evaluated using Pearson’s correlation coefficient (*r*). To evaluate the proportion of variability in the BICR estimates explained by the LE estimates, the coefficient of determination (*R*^2^) was calculated from a linear regression model based on log(HR) weighted by the sample size. In addition, the magnitude of agreement between BICR and LE for PFS was quantified by


$$\begin{array}{l} Hazard\;ratio\;ratio:HRR=\frac{HR_{BICR}}{HR_{LE}} \end{array}$$

where HR_BICR_ and HR_LE_ are the BICR- and LE-based PFS HRs.^4^ A random effects model, weighted by the comparisons’ sample size, with log(HR) as the response variable, indicator of LE vs. BICR as a fixed effect, and with comparison as a random effect, was used to estimate the overall hazard ratio ratio (HRR) and its 95% CI across the comparisons. Additionally, subgroup analyses were conducted for double-blind and open-label comparisons to investigate the impact of masking on the study results.

HRRs were also calculated on an individual comparison level and grouped into four intervals: ≤0.85; (0.85, 1]; (1, 1.15]; and >1.15. These intervals were further analyzed by key study characteristics (eg, masking, phase) to understand the relationships between these characteristics and HRR.

When a protocol prespecified that a PFS comparison should be formally tested the statistical inference was classified as “statistically significant” or “not statistically significant” based on its *P*-value crossing the prespecified α-boundary or not. A two-way contingency table was generated to evaluate the consistency of the statistical inference between BICR and LE, and Cohen’s kappa coefficient was calculated.

### ORR Meta-Analysis

Similar to the PFS meta-analysis, Pearson’s correlation coefficient (*r*) and the coefficient of determination (*R*^2^) were calculated between BICR and LE on log(OR of ORR). Furthermore, the magnitude of agreement between BICR and LE for ORR was quantified by


$$\begin{array}{l} \begin{array}{lll} & Odds\;ratio\;ratio\;of\;the\;response:\;OddsRR=\frac{OR_{BICR}}{OR_{LE}}\; & where,\;OR_{BICR}=\frac{ORR_{BICR}^{arm\;A}(1-ORR_{BICR}^{arm\;B})}{ORR_{BICR}^{arm\;B}(1-ORR_{BICR}^{arm\;A})},\;OR_{LE}=\frac{ORR_{LE}^{arm\;A}(1-ORR_{LE}^{arm\;B})}{ORR_{LE}^{arm\;B}(1-ORR_{LE}^{arm\;A})}\; \end{array} \end{array}$$

A similar random effects model as used in the PFS meta-analysis was applied to estimate the odds ratio ratio (OddsRR) and its 95% CI across all comparisons and for double-blind and open-label comparisons separately.

## Results

Table 1 summarizes the characteristics of the investigated comparisons.

**Table 1.** Overview of characteristics of BICR vs. LE comparisons.

Characteristics	PFS comparisons (*n* = 55)	ORR comparisons (*n* = 40)
Masking		
Double-blind	17 (30.9%)	7 (17.5%)
Open-label	38 (69.1%)	33 (82.5%)
Sample size		
Mean	631	625.9
Min-max	80-1904	80-1904
Phase		
III	48 (87.3%)	33 (82.5%)
I/II	7 (12.7%)	7 (17.5%)
Randomization ratio		
1:1	43 (78.2%)	34 (61.8%)
2:1	12 (21.8%)	6 (10.9%)
Clinical cut-off date		
Before 2015	24 (43.6%)	18 (45.0%)
On or after 2015	31 (56.4%)	22 (55.0%)
Sponsor		
Roche	50 (90.9%)	38 (95.0%)
Cooperative group	5 (9.1%)	2 (5.0%)
Criterion		
RECIST/(n)Lugano	43 (78.2%)	33 (82.5%)
Other	12 (21.8%)	7 (17.5%)

Abbreviations: BICR, blinded independent central review; LE, local evaluation; ORR, objective response rate; PFS, progression-free survival; RECIST, Response Evaluation Criteria in Solid Tumours.

### Progression-Free Survival

**Figure 1.**
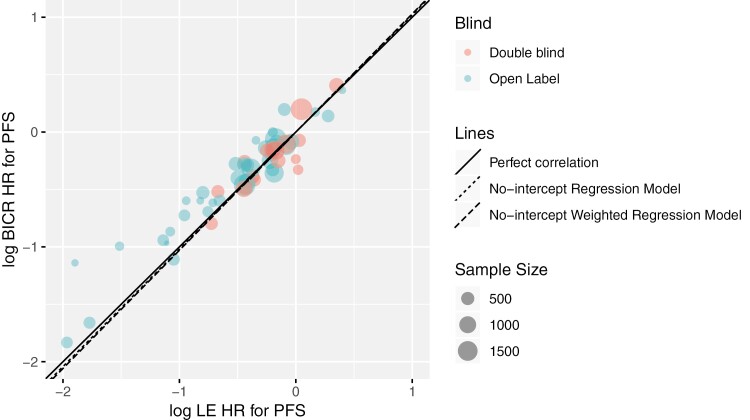
BICR vs. LE log(PFS HR) by blinding status of trial. Abbreviations: BICR, blinded independent central review; HR, hazard ratio; LE, local evaluation; PFS, progression-free survival.

A high correlation between log(HR_BICR_) and log(HR_LE_) of PFS is illustrated in Fig. 1. Each circle represents one comparison, and the size of the circle is proportional to the sample sizes of the corresponding comparison. The solid line is the reference line denoting perfect correlation. The circles scatter closely to the reference line, indicating an overall high agreement between the log(HR_BICR_) and log(HR_LE_). The circles for the double-blind comparisons (red) are close to and spread on both sides of the solid line, regardless of the sample size, indicating that there is no systematic over- or under-estimation of the treatment effect by BICR vs. LE assessment. The circles for the open-label comparisons (blue) tend to be above the solid line, indicating a potential bias favoring the experimental arm by LE compared with BICR. This is particularly notable for open-label comparisons with smaller sample sizes (small blue circles). The open-label comparisons with larger sample sizes (large blue circles) are closer to the solid line, indicating higher agreement and less bias.

Table 2 presents the agreement between BICR and LE PFS results. A high degree of correlation between log(HR_BICR_) and log(HR_LE_) was shown with Pearson’s correlation coefficient of *r* = 0.946 (95% CI, 0.909, 0.968) in the overall analysis including 55 pairwise comparisons. Subgroup analyses in the double-blind (*n* = 17) and open-label (*n* = 38) comparisons showed consistent results with the overall analysis. The degrees of association were 0.882 (95% CI, 0.697, 0.957) and 0.956 (95% CI, 0.916, 0.977) in the double-blind and open-label groups, respectively. In the overall analysis, 89.3% (*R*^2^ = 0.893 [95% CI, 0.802, 0.942]) of the variability in the log(HR_BICR_) can be explained by the weighted linear regression model with log(HR_LE_) as the explanatory variable, which again demonstrates the high agreement between BICR and LE. The estimated overall HRR from the random effects model was 1.044 (95% CI: 1.009, 1.081), indicating an average difference of only 4.4% between HR_BICR_ and HR_LE_. The estimated HRR in the open-label group was numerically slightly higher than that in the double-blind group (1.062 vs. 1.014), but both were close to 1 indicating a high degree of agreement in the PFS HR estimates overall.

**Table 2.** Agreement assessment of PFS between BICR and LE.

Number of comparisons	Log(HR)	Log(HR)	HRR
	*r* (95% CI)^a^	*R* ^2^ (95% CI)^b^	(95% CI)^c^
Overall: 55	0.946 (0.909, 0.968)	0.893 (0.802, 0.942)	1.044 (1.009, 1.081)
Double-blind: 17	0.882 (0.697, 0.957)	0.858 (0.643, 0.949)	1.014 (0.958, 1.073)
Open-label: 38	0.956 (0.916, 0.977)	0.904 (0.803, 0.956)	1.062 (1.016, 1.110)

^a^ *r*: Pearson’s correlation coefficient between log(HR_BICR_) and log(HR_LE_).

^b^ *R* ^2^: Coefficient of determination from weighted linear regression model on log(HR), weighted by sample size.

^c^HRR: Hazard ratio ratio = HR_BICR_/HR_LE_, estimated from linear mixed effect model, weighted by sample size.

Abbreviations: BICR, blinded independent central review; HR, hazard ratio; LE, local evaluation; PFS, progression-free survival.

To further understand the level of agreement at the individual trial level, HRRs of each comparison were calculated and summarized by intervals (Table 3). Among the total of 55 randomized comparisons investigated, 63.6% (35/55) had 0.85 <HRR ≤1.15, indicating a reasonable degree of agreement between HR_BICR_ and HR_LE_. For the remaining 5.5% (3/55) and 30.9% (17/55) of the comparisons, BICR estimated a ≥15% stronger or a >15% weaker treatment effect compared to the LE estimate, respectively. No meaningful difference in the distribution of HRRs was observed comparing trials run before 2015 vs. in or after 2015 nor comparing trials sponsored by Roche vs. by cooperative groups. However, we observed that HRRs were more skewed towards the >1.15 interval in the open-label group than in the double-blind group (36.8% [14/38] vs. 17.6% [3/17]) and in the 2:1 randomization group than in the 1:1 randomization group (50% [6/12] vs. 25% [11/43]). This indicates that an HRR greater than 1.15 is more likely in open-label trials than in double-blind trials and with a randomization ratio of 2:1 than with a ratio of 1:1. Additionally, comparisons with HRR >1.15 had relatively smaller sample sizes (median 351) compared with trials with HRR closer to 1 (median 651 for HRR (0.85, 1] and 636 for HRR (1, 1.15]).

**Table 3.** BICR vs. LE comparisons by PFS HRR intervals.

Characteristics	*n*	HRR^a^			
		≤0.85	(0.85, 1]	(1, 1.15]	>1.15
Overall, *n* (%)	55	3 (5.5%)	17 (30.9%)	18 (32.7%)	17 (30.9%)
Masking					
Double-blind	17	2 (11.8%)	8 (47.1%)	4 (23.5%)	3 (17.6%)
Open-label	38	1(2.6%)	9 (23.7%)	14(36.8%)	14 (36.8%)
Sample size					
Median	503	255	651	636	351
Min-max	80-1904	222-1400	121-1873	152-1873	80-1904
Rand. Ratio					
1:1	43	1 (2.3%)	14(32.6%)	17 (39.5%)	11(25.6%)
2:1	12	2 (16.7%)	3 (25.0%)	1(8.3%)	6(50.0%)
Time					
Before 2015	24	1(4.1%)	8 (33.3%)	8 (33.3%)	7 (29.2%)
In or after 2015	31	2 (6.5%)	9 (29.0%)	10 (32.3%)	10 (32.3%)
Sponsor					
Roche	50	3 (6.0%)	16 (32.0%)	16 (32.0%)	15 (30.0%)
Cooperative group	5	0	1 (20.0%)	2 (40.0%)	2 (40.0%)

^a^HRR: Hazard ratio ratio = HR_BICR_/HR_LE_, estimated from linear mixed effect model, weighted by sample size

Abbreviations: BICR, blinded independent central review; HR, hazard ratio; LE, local evaluation; PFS, progression-free survival.

Table 4 summarizes the consistency of statistical inferences between BICR and LE based on PFS results. In the remainder of the text, we will denote a statistically significant PFS difference based on BICR assessment as “BICR+” and a not statistically significant difference as “BICR-“; “LE+” and “LE−“ are analogously defined for the LE-based assessment. Forty-six of the 55 comparisons were alpha-controlled leading to statistical inferences. For the majority (87.0% [40/46]) of the 46 comparisons, BICR agreed with LE in terms of the resulting statistical inferences: 37.0% (17/46) of the comparisons led to a BICR+/LE+ result and 50.0% (23/46) to a BICR−/LE- result. Discordant results, i.e., BICR+/LE− and BICR−/LE+ combinations, were observed in 1 (2.2%) and 5 (10.9%) of the comparisons, respectively. Fifteen of the 17 comparisons with HRR >1.15 (Table 3) were formally tested, 13 of these had concordant and 2 had discordant BICR/LE results. Cohen’s Kappa was 0.737 indicating a substantial agreement between BICR and LE. The impact of BICR in these inconsistent cases on the regulatory submission activities is described in the Discussion section.

**Table 4.** Consistency of statistical inference per PFS among α-controlled comparisons.

LE, *N* = 46	BICR	
	Statistically significant (BICR+)	Not statistically significant (BICR−)
Statistically significant (LE+)	17 (37.0%)	5 (10.9%)
Not statistically significant (LE−)	1 (2.1%)	23 (50.0%)

Statistical inference (statistically significant vs. not statistically significant) was based on whether the protocol pre-specified boundary of the formal hypothesis testing for PFS was crossed or not.

Abbreviations: BICR, blinded independent central review; LE, local evaluation; PFS, progression-free survival.

Overall, a high agreement between BICR and LE estimates of the PFS treatment effect was observed in the meta-analysis, while the agreement was slightly stronger in the double-blind subgroup than in the open-label group. At the individual-trial level, BICR and LE gave consistent statistical inferences in the majority of the comparisons, which is in line with the meta-analysis result.

### Objective Response Rate

ORR was less commonly used as a primary endpoint in pivotal studies, thus, fewer BICR results for ORR were available from such studies (*n* = 40), especially from double-blind studies. There are only 7 comparisons in the double-blind subgroup, therefore, the comparisons between the double-blind and open-label subgroups should be interpreted with caution.

**Figure 2.**
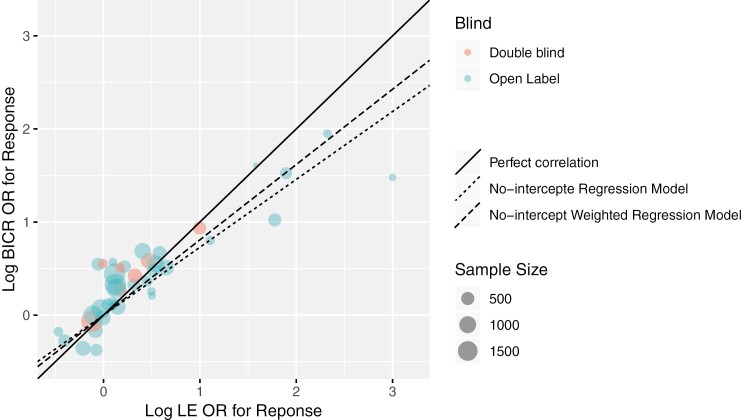
BICR vs. LE log(OR of ORR) by blinding status of trial. Abbreviations: BICR, blinded independent central review; LE, local evaluation; OR, odds ratio; ORR, objective response rate.

The scatter plot of log(OR_BICR_) vs. log(OR_LE_) is provided in Fig. 2. Compared to Fig. 1, the circles for log(OR) are more dispersed from the solid reference line of perfect correlation than those for log(HR). Overall, the results are more variable than for PFS. More circles are above the solid line, indicating larger treatment benefit differences in the experimental arm over the control arm are estimated by BICR compared with those by LE. Some smaller circles are further away from the solid line, indicating that the difference between LE and BICR is larger in comparisons with smaller sample size. Due to the small number of double-blind cases, there is no clear pattern comparing the double-blind vs. open-label subgroups.

Analogous analyses as conducted for PFS were performed for ORR (Table 5). A high correlation (*r* = 0.890 [95% CI, 0.801, 0.941]) between log(OR_BICR_) and log(OR_LE_) was observed, though it was numerically lower than that for PFS log(HR). 76.7% (*R*^2^ = 0.767 [95% CI, 0.631, 0.874]) of the variability in log(OR_BICR_) can be explained by the weighted linear regression model with log(OR_LE_) as the explanatory variable. The estimated overall OddsRR from the random effects model was 1.065 (95% CI: 0.983, 1.154), indicating an average of 6.5% difference between OR_BICR_ and OR_LE_ for response. Subgroup analyses in the double-blind and open-label comparisons generally showed consistent results with the overall analysis with largely overlapping CIs.

**Table 5.** Agreement assessment of OR between BICR and LE.

Number of comparisons	Log(OR)	Log(OR)	OddsRR
	*r* (95% CI)^a^	*R* ^2^ (95% CI)^b^	(95% CI)^c^
Overall: 40	0.890 (0.801, 0.941)	0.767 (0.631, 0.874)	1.065 (0.983, 1.154)
Double-blind: 7	0.826 (0.194, 0.974)	0.868 (0.098, 0.997)	1.108 (0.978, 1.257)
Open-label: 33	0.900 (0.806, 0.950)	0.760 (0.603, 0.880)	1.055 (0.959, 1.162)

^a^ *r*: Pearson’s correlation coefficient between log(OR_BICR_) and log(OR_LE_).

^b^ *R* ^2^: Coefficient of determination from weighted linear regression model on log(OR), weighted by sample size.

^c^OddsRR: Odds ratio ratio for response = log(OR_BICR_)/log(OR_LE_), estimated from random effects model, weighted by sample size.

Abbreviations: BICR, blinded independent central review; LE, local evaluation; OR, odds ratio; ORR, objective response rate.

In summary, although the degree of agreement between log(OR_BICR_) and log(OR_LE_) was less strong compared to that for PFS log(HR) the results are generally in line with the PFS results, and a high degree of agreement between BICR and LE ORR results was observed. Due to the limited number of comparisons in the double-blind subgroup, no robust comparison to the open-label subgroup can be made.

## Discussion and Conclusion

We conducted two meta-analyses of 49 Roche-supported randomized clinical trials from 2006 to 2020 in solid tumors and hematologic malignancies that reported both BICR- and LE-assessed results for PFS and/or ORR. This dataset is devoid of selection bias as all trials that matched these criteria were included in the analysis. Furthermore, this dataset is unique in that all studies meet the same Roche standards, ensuring a consistent approach for data quality checks, and a requirement of completeness of the data, and additional information such as the resulting statistical inference and regulatory submission decision is available. Similar published analyses, however, used public sources such as literature searches, conference abstracts,^6-10^ or FDA and EMA review documents from various sponsors,^4^ which may be more heterogeneous and subject to reporting bias.^11^ Their analyses included only solid malignancies, whereas our work also included hematologic malignancies. It has been criticized^11^ that a substantial proportion of the LE-BICR comparisons used in other meta-analyses^4,6^ may not be sufficiently independent because in some trials, blinded tumor assessments were provided to the BICR, and in others, BICR was described as confirming LE progression. None of this applies to the comparisons included in our meta-analyses; the only clinical information shared between both assessor groups was strictly defined in the imaging charter including, eg, information about radiotherapy, surgery, or biopsy results. Some authors^7-10^ also examined the impact of the numerical differences in the treatment effect estimates on the concordance of the statistical inference. However, in our work we additionally assessed the impact of discordant inferences on the decision to pursue a regulatory submission. Overall, we believe that this aspect provides valuable novel insights and that the specific properties of our clinical trial dataset address several shortcomings of previous, similar analyses.

We acknowledge that we restricted our subgroup analyses to the masking type because the limited number of trials did not allow us to study important differences in other interesting characteristics (eg, tumor types and disease criteria). Studies with multiple comparisons included in our analysis were analyzed as independent comparisons. The correlation between such comparisons was not taken into account. In addition, we did not analyze patient-level concordance/discordance (ie, early/late discrepancy rate) nor other measures beyond PFS HR and ORR (eg, median PFS). In most of the studies in our analysis, BICR was pre-specified, the knowledge about which may have reduced LE bias to some degree. Lastly, our work only reflects the experience with Roche-supported studies, where the setting and monitoring are relatively homogeneous, and their associated processes may not be transferable to other settings.

### Consistency and Bias Between BICR and LE Treatment Effect Estimates

Overall, a high level of agreement between HR_BICR_ and HR_LE_ PFS estimates was found, irrespective of trial masking. On average, the observed bias was small: A HRR of 1.044 (95% CI, 1.009, 1.081) was observed between HR_BICR_ and HR_LE_ for PFS, with BICR estimating a weaker treatment effect; this is in line with findings from other authors.^4,6,7,9^ Our results also showed that while there was no evidence of systematic bias in double-blind comparisons, the HRR was observed to be more often greater than one for open-label comparisons, particularly with smaller sample sizes, indicating that HR_BICR_ is less likely to favor experimental treatment arms than HR_LE_ in these trials. In about two-thirds of the comparisons, LE overestimated the treatment effect compared to BICR by not more than 15% or even underestimated it. In the third of comparisons with a stronger LE overestimation (more than 15% over BICR), we found a two-fold relative overrepresentation of open-label comparisons.

### Impact on Decision Making: Statistical Inference and Regulatory Submission Activities

In addition to showing high agreement between BICR and LE estimates, our results showed consistent statistical inferences in 87.0% (40/46) of the comparisons of PFS. Furthermore, for the comparisons with HRR >1.15, 86.7% (13/15) yielded consistent statistical inferences. This finding is in line with a meta-analysis from 2018,^9^ which showed a consistency of 78%. The high consistency of the statistical inferences between BICR and LE PFS reflects the high reliability of LE results in the majority of the comparisons.

For each of the 6 comparisons that yielded discordant BICR- and LE-based statistical inferences, their subsequent regulatory submission activities were assessed. For the one study with BICR+/LE− results, no regulatory submission was pursued, demonstrating that the BICR+ results did not “rescue” the LE− primary endpoint results. Two of the five cases had BICR−/LE+ results and were filed to a regulatory authority and approved based on their primary LE+ and clinically meaningful results, showing that the BICR results did not change the key conclusions from the LE primary endpoint results. The remaining 3 cases had BICR−/LE+ results and were not submitted for filing based on the overall benefit-risk assessment considering the totality of the data. The totality of data included borderline, not clinically meaningful benefit based on LE, lack of strong supportive OS results, as well as added safety burden. This demonstrates that the BICR dataset itself was not the key driver for the regulatory submission decision.

Overall, this analysis shows that the BICR itself did not significantly impact the study interpretation nor drive the regulatory submission decisions for the Roche-supported studies investigated.

### BICR Challenges

Traditionally, BICR was mainly performed in late-stage open-label trials. More recently, however, we observe an increasing tendency of requests for BICR from Health Authorities.

Intended to mitigate bias in local site evaluations, BICR-based analyses are still susceptible to bias themselves because of informative censoring,^12-16^ which occurs when tumor assessments stop once LE progression is determined. As a result, BICR-determined progression cannot occur after LE progression. Patients with LE-determined progression but not agreed by BICR are censored in the BICR analysis, which may lead to larger median PFS estimates, biased HR estimates, and loss of power. Real-time BICR and confirmation of progression during the trial may help alleviate this bias, however, real-time BICR implementation has practical, ethical, and legal hurdles.

In practice, some clinical data (besides imaging) are available to LE, but not to BICR. In certain cases, these additional data such as physical examinations and symptom assessments may help the investigator to obtain a more comprehensive picture of the patient’s disease status. This limits the value of BICR in such cases.

In event-driven trials with BICR-based primary endpoints, any delay in imaging data submission or BICR reading can lead to issues in PFS event tracking making it difficult for the sponsor to exactly match the pre-determined number of events. This can have an adverse impact on the statistical power and timely delivery of the study results for regulatory submissions.

### Recommendations

In our analysis, we showed that when HR_BICR_ and HR_LE_ were not concordant, BICR results alone did not change the interpretation of the study outcome nor drive the regulatory submission decision. Hence, reevaluation of the need for BICR for specific study designs in future oncology trials is warranted. We recommend assessing the potential for bias per LE on a study-by-study basis and limiting BICR to the studies with an increased likelihood of bias of the treatment effect estimate.

In a truly double-blinded study, the potential for evaluation bias that overestimates the treatment effect is eliminated.^13,17^ Considering the results of our analysis, we recommend a prospective assessment of sources of potential variability and the risk of bias for each new double-blind trial to decide if BICR is scientifically justified in the specific study setting. If the risk of bias is anticipated to be low, e.g., when the disease is not clinically symptomatic, the toxicity profile of the tested drug is not going to effectively unblind the study, or when established tumor assessment criteria are applied, we advocate that there is no need for a BICR in the trial.

For trials with a higher potential risk of bias, confirmation of LE results by BICR is likely helpful to minimize bias. This includes open-label trials, double-blind randomized trials in clinically symptomatic diseases, trials with a characteristic toxicity profile of the tested drug, and trials in which new or complex tumor assessment criteria are applied. In these cases, we propose BICR as a sensitivity analysis. Sensitivity analyses target the same estimand as the primary analysis. This means there is usually no change to the treatment, population, variable, handling of intercurrent events, and population-level summary measure.^18^ These analyses assess different assumptions of the statistical methods to explore the robustness of the treatment effect estimate.^19^ The estimator is the same for the LE and the BICR analysis, however, the assumptions are different; therefore, BICR is a sensitivity analysis of LE. For the sensitivity analysis, it can be decided on a case-by-case basis if a full BICR or an audit sample analysis is conducted.

An audit approach, in which only a sample of the radiographic images is sent for BICR, is an alternative to performing the full BICR.^13,14,20,21^ It may partially alleviate the burden of a full BICR. However, this approach has not been widely adopted in practice due to practical challenges including requiring a sufficiently large audit sample for stable audit assessment, the timing of audit sample assessment (which may delay the overall timeline), selection between available methods, and uncertainties in the results derived from the sample dataset.

Using LE-based assessments as primary evidence and adding BICR as a sensitivity analysis only when scientifically justified based on a study-by-study assessment will help expedite delivering novel treatments to patients at a reduced cost.

Regardless of whether or when BICR is performed, we recommend the collection and storage of radiographic images acquired during the study conduct. This allows for an “on demand” BICR if deemed critical at a later stage, or it can be used to support future exploratory purposes. Furthermore, the prospective collection of all images might also mitigate potential LE bias knowing that a BICR is potentially planned.

## Data Availability

The data underlying this article will be shared on reasonable request to the corresponding author.
